# Polymer blend lithography for metal films: large-area patterning with over 1 billion holes/inch^2^

**DOI:** 10.3762/bjnano.6.123

**Published:** 2015-05-26

**Authors:** Cheng Huang, Alexander Förste, Stefan Walheim, Thomas Schimmel

**Affiliations:** 1 Institute of Applied Physics and Center for Functional Nanostructures (CFN), Karlsruhe Institute of Technology (KIT), 76128 Karlsruhe, Germany; 2 Institute of Nanotechnology (INT), Karlsruhe Institute of Technology (KIT), 76021 Karlsruhe, Germany

**Keywords:** localized surface plasmonic resonance, metal islands, metal nanostructures, metal polymer blend lithography (metal PBL), nano-patterned template, nanoscale discs, optical transmission, perforated metal film, polymer phase separation, poly(methyl methacrylate) (PMMA), polystyrene (PS), self-assembly, spin-coating, surface plasmons

## Abstract

Polymer blend lithography (PBL) is a spin-coating-based technique that makes use of the purely lateral phase separation between two immiscible polymers to fabricate large area nanoscale patterns. In our earlier work (Huang et al. 2012), PBL was demonstrated for the fabrication of patterned self-assembled monolayers. Here, we report a new method based on the technique of polymer blend lithography that allows for the fabrication of metal island arrays or perforated metal films on the nanometer scale, the metal PBL. As the polymer blend system in this work, a mixture of polystyrene (PS) and poly(methyl methacrylate) (PMMA), dissolved in methyl ethyl ketone (MEK) is used. This system forms a purely lateral structure on the substrate at controlled humidity, which means that PS droplets are formed in a PMMA matrix, whereby both phases have direct contact both to the substrate and to the air interface. Therefore, a subsequent selective dissolution of either the PS or PMMA component leaves behind a nanostructured film which can be used as a lithographic mask. We use this lithographic mask for the fabrication of metal patterns by thermal evaporation of the metal, followed by a lift-off process. As a consequence, the resulting metal nanostructure is an exact replica of the pattern of the selectively removed polymer (either a perforated metal film or metal islands). The minimum diameter of these holes or metal islands demonstrated here is about 50 nm. Au, Pd, Cu, Cr and Al templates were fabricated in this work by metal PBL. The wavelength-selective optical transmission spectra due to the localized surface plasmonic effect of the holes in perforated Al films were investigated and compared to the respective hole diameter histograms.

## Introduction

Research on micro-/nano-sized island arrays and perforated films has drawn wide interest due to their applications in various fields, such as optical devices [1–2], DNA or protein electrophoresis [3–4], and catalysis [5–6]. Varieties of techniques have been developed to achieve metal or semiconductor nanopatterns, such as electron beam lithography [7–9], nanosphere lithography [10–13], laser interference lithography [14–15], AFM-based dip-pen lithography [16], and more. Masuda and his colleagues used anodic porous alumina as lithographic mask for the fabrication of both ordered gold or silver nanodot arrays and ordered nanohole arrays in a gold film [17–18]. Lithographic methods incorporated with self-assembled block copolymers are also promising ways to fabricate sub-100 nm metal nanodots or perforated films, however these processes are often complicated or time consuming, e.g., as vapor annealing for block-copolymers takes days to complete and UV radiation or reactive ion etching (RIE) is required for the lift-off process [19–20].

Here, we introduce a very rapid and cost-effective way to fabricate metal island arrays or perforated metal films via metal polymer blend lithography (metal PBL). The polymer blend lithography was applied in our earlier reports to fabricate self-assembled monolayer (SAM) patterns (monolayer PBL) [21], which were used, e.g., for the selective growth of ZnO nanostructures [22]. Instead of silicon wafers, other materials such as glass or metal can also be used as substrates. For example iron was used as substrate for the fabrication of nanoporous gold mesoflower arrays, which served afterwards for surface-enhanced Raman scattering [23]. With the metal PBL technique it is possible to fabricate more than one billion metal islands or holes on a substrate with a size of 1 inch × 1 inch.

## Results and Discussion

### Metal polymer blend lithography (metal PBL) for metal islands or perforated films

**Figure 1:**
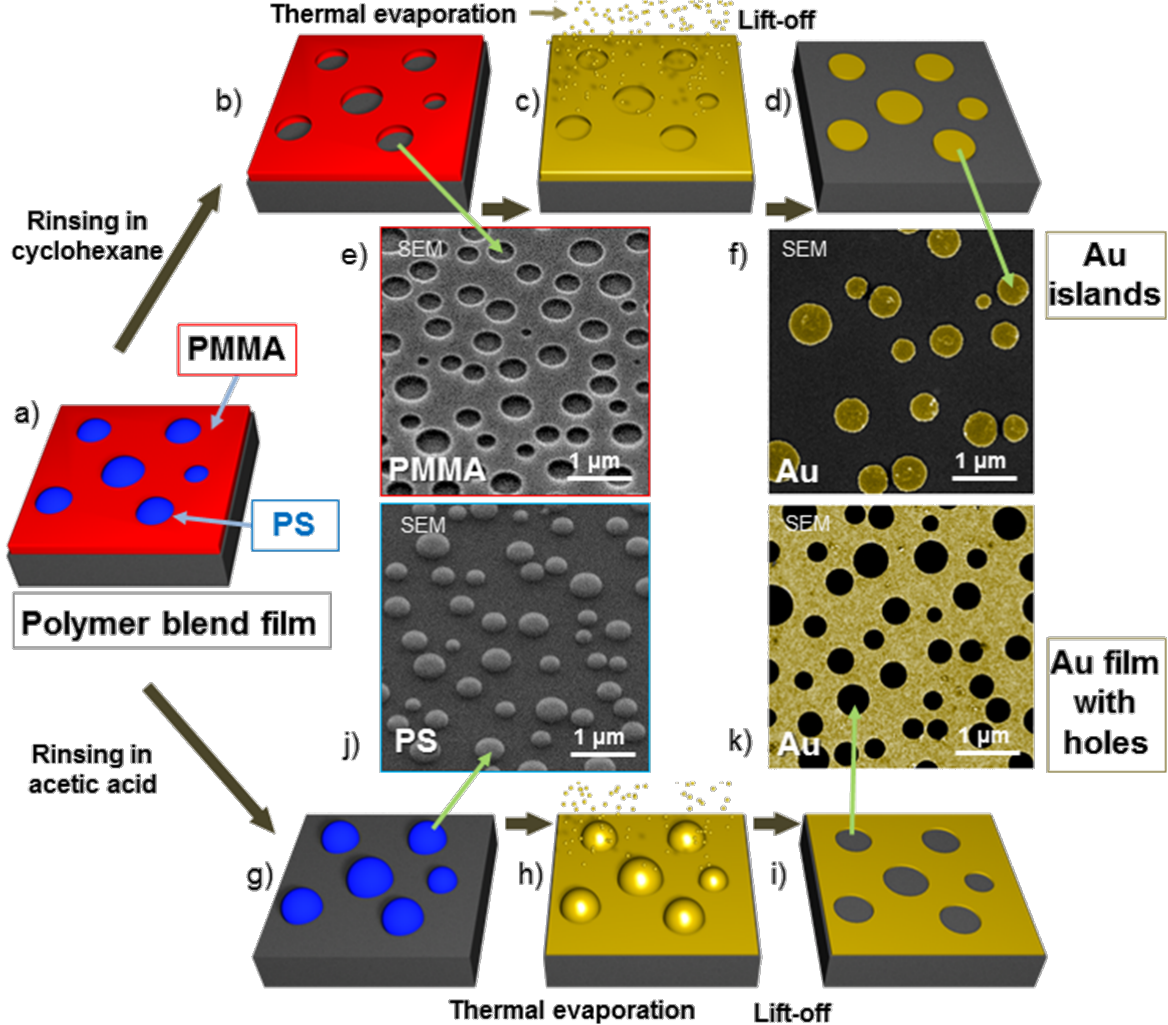
Schematic drawing and experimental results of the metal PBL process for the fabrication of metal nano-patterns. a) A blend film (PS/PMMA 3:7 mass ratio) is formed through spin-coating. PS droplets are formed in a PMMA matrix, and both phases have contact to the substrate as well as to the air. b) PS droplets are selectively dissolved by cyclohexane. c) Deposition of metal via thermal evaporation. d) After lift-off via snow-jet treatment, a negative metal copy of the PMMA mask is formed. e) SEM image of a perforated PMMA film (taken at a tilting angle of 45°). f) SEM image of gold islands. g) The PMMA matrix is selectively dissolved by acetic acid. h) Deposition of metal via thermal evaporation. i) After lift-off via snow jet, a negative metal copy of the PS mask is formed. j) SEM image of PS droplets (taken at a tilting angle of 45°). k) SEM image of a perforated gold film.

The metal polymer blend lithography method (metal PBL) is schematically demonstrated in Figure 1. The spin casting of the polystyrene (PS)/poly(methyl methacrylate) (PMMA) blend film from a blend solution ends with a purely lateral phase separation between the two immiscible polymers under controlled spin-coating parameters (see Figure 1a). As reported in our earlier publication, the ambient atmosphere (humidity), the molar mass of the polymers and the mass ratio between the two polymers in the blend solution play important roles in the determination of the structure of the formed films [21,24]. In this study cyclohexane was used as the selective solvent for removing PS while acetic acid for removing PMMA. Via selective dissolution, either the PMMA matrix (see Figure 1b, PMMA marked in red) or the PS droplets (see Figure 1g, PS marked in blue) can be kept on the substrate for the lithographic application.

After the deposition of metal by thermal evaporation, the desired metal covers the whole surface of the sample (see in Figure 1c and Figure 1h, metal is marked in gold). Finally the snow jet method is applied for the lift-off of the polymer mask and a nano-patterned metal structure (either a perforated film as shown in Figure 1i or islands as shown in Figure 1d) remains on the substrate. This metal pattern is an exact replica of the selectively dissolved polymer.

Figure 1e,f,j,k show the SEM images of the polymer lithographic masks and the gold islands together with the perforated gold film respectively, which were fabricated applying these masks. The density of the holes shown in Figure 1k is about 130 million/cm^2^. Figure 1e is an SEM image taken at a tilting angle of 45° of a polymer lithographic mask, which is schematically shown in Figure 1b. After the polymer blend film was rinsed in cyclohexane, the PS droplets were dissolved and a perforated PMMA film with holes was left on the silicon substrate. The diameters of these holes range from 200 to 800 nm. The metal islands deposited into the holes are stable and resist the snow-jet treatment that is used for the lift-off of the PMMA mask. For some metals such as gold and silver, an adhesion enhancing interlayer of metal, e.g., titanium or chromium is necessary between the desired metal and the substrate. The SEM top view image of the gold islands with a thickness of 20 nm is shown in Figure 1f. The islands have the same size (200–800 nm) as the holes in the polymer film (see Figure 1e).

**Figure 2:**
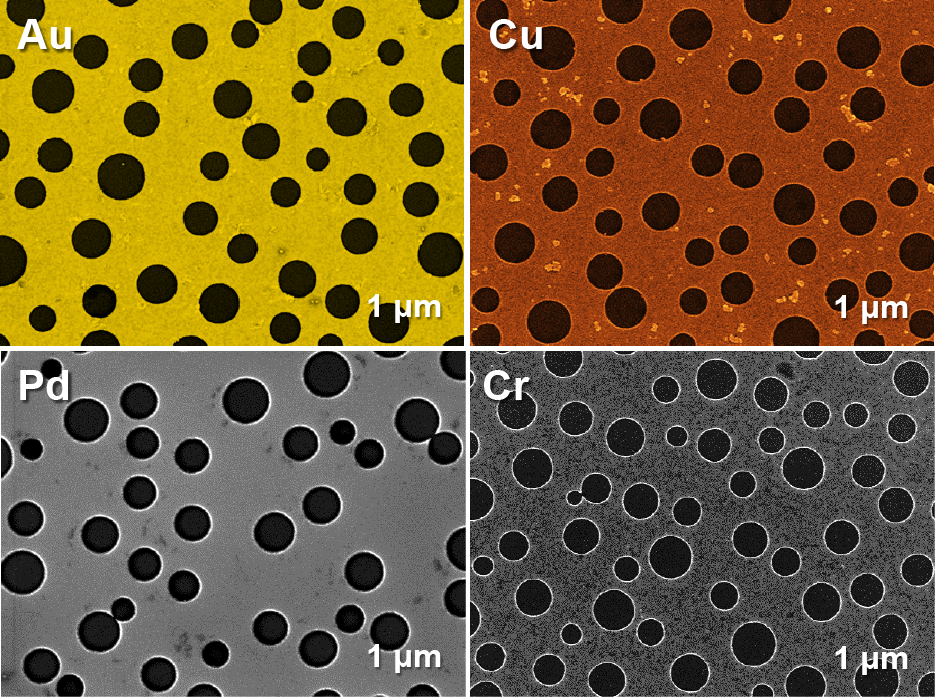
SEM images of perforated Au, Pd, Cu, and Cr films fabricated by metal PBL. Aluminum films are shown below in Figure 4.

For the fabrication of a perforated metal film, the polymer blend must be rinsed in acetic acid instead of cyclohexane. In this case, consisting PS droplets is left on the substrate. A smooth and clean silicon surface can be seen in the SEM image taken at a tilting angle of 45°. The “Go stone”-like PS droplets are ideal for the following lift-off process (see Figure 1j). Consequently the fabricated perforated gold film (see Figure 1k) has holes with perfectly round shapes and smooth edges. The diameters of these holes range also from 200 to 800 nm, which is in accordance with the PS lithographic mask. To demonstrate the feasibility of the reported lithographic technique to form patterns of various metals, we used the process described in Figure 1g–i to fabricate 20 nm thick films of gold, palladium, copper and chromium (see Figure 2).

### Sub-100 nm holes and islands

**Figure 3:**
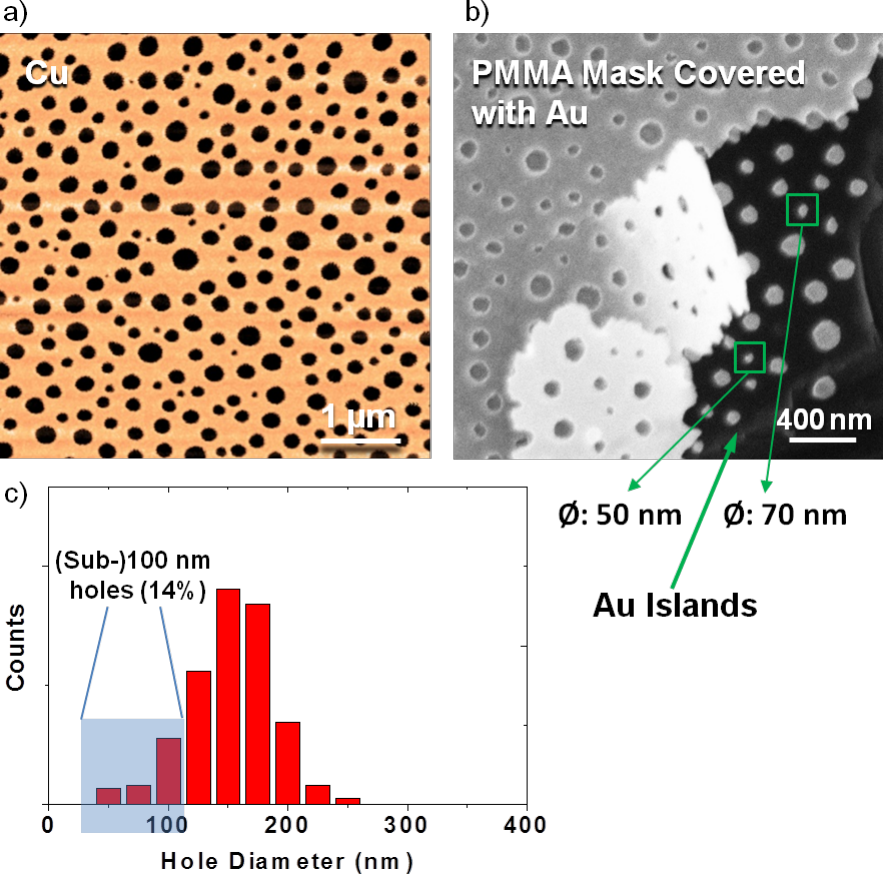
Sub-100 nm holes in a Cu film (left) and gold islands (right) fabricated by metal PBL. a) AFM image of a perforated Cu film with a thickness of 20 nm. b) SEM image of gold islands (with a thickness of 20 nm) with PMMA lithographic mask covered with gold. c) Diameter distribution of the holes in Figure 3a. The holes have an average diameter of about 150 nm and there are holes found in the (sub-)100 nm range (about 14%).

By using polystyrene with very low molecular weight (9.58 kg/mol), it is possible to obtain a polymer blend film with very small PS droplets. This offers the possibility to fabricate sub-100 nm metal islands or sub-100 nm holes in metal films, which is comparable, e.g., to the size of viruses. Figure 3a is an AFM image of a perforated Cu film with a thickness of 20 nm. The diameters of the holes range from 50 to 250 nm and have an average size of about 150 nm (see Figure 3c). The (sub-)100 nm holes are homogeneously distributed over the samples (about 14%). Figure 3b is an SEM image of the gold islands together with the PMMA lithographic mask, which was also covered with gold. The gold islands have statistically the same size as the holes in Figure 3a. In general, metal PBL is a good supplementary to the conventional lithographic methods for fabrication of structures in hundreds of nanometer or even sub-100 nanometer range in a versatile and up-scalable way.

### Localized surface plasmonic resonance of perforated Al films

The size of the holes in the fabricated perforated metal films is in the range of the wavelength of visible light. Therefore it is interesting to investigate their optical transmission properties. It has been published in 2003 by Barnes et al. that ordered hole arrays fabricated by e-beam lithography can be used as wavelength-selective optical filters [25]. The photons couple to surface plasmons on the incident side of a nanohole film. These surface plasmons convert back to photons after they propagate through the holes to the opposite side of the film [26]. In recent years, also the colloidal lithography method has been used to study the surface plasmonics of random nano-hole arrays in metal films [26–27].

**Figure 4:**
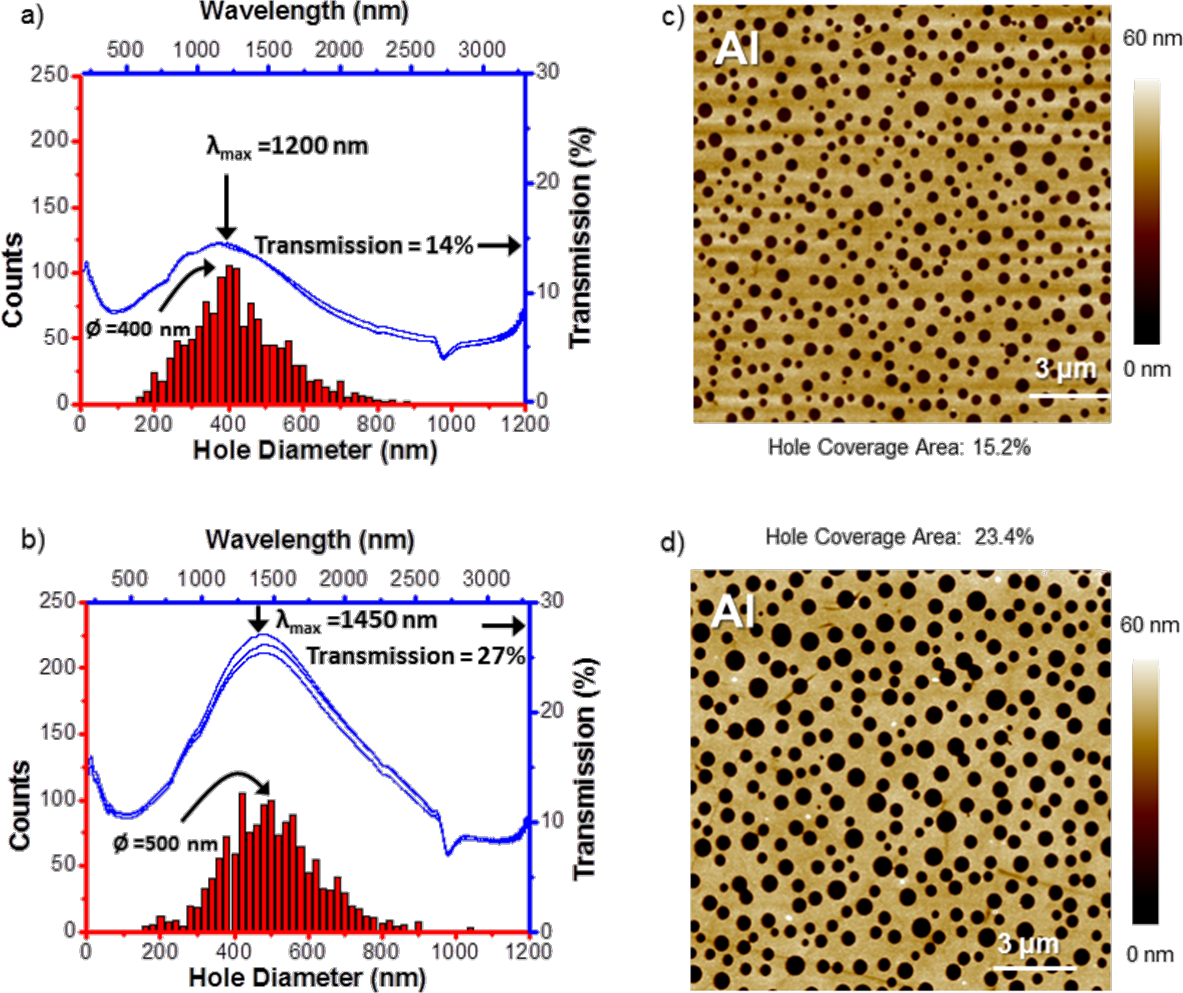
Optical transmission due to localized surface plasmonic resonance (blue) of perforated aluminum films with two different hole size distributions (red). Top: smaller holes (PS/PMMA mass ratio 3:7) with a hole coverage area of 15.2%, bottom: larger holes (PS/PMMA mass ratio 4:6) with a hole coverage area of 23.4%. a) and b): The hole size distributions are shown with red columns, the data are the summarization of five 15 μm × 15 μm AFM images taken at randomly selected positions. The optical transmission spectra, taken at various sample positions, are plotted as blue line graphs. The representative AFM topography (tapping mode) of the samples is shown in c) and d).

Since the skin depth, and therefore the transmission of thin films, of Cu, Au and Ag is relatively high, we selected aluminum, which shows a high reflectivity in the range of 220 to 650 nm (about 90%) already at a thickness below 30 nm [28]. Our result, using templates produced according to the process described in Figure 1g–i is shown in Figure 4. A transmission difference of 13% can be observed (see Figure 4a,b) due to the hole coverage area difference between the two samples (see Figure 4c,d). A red shift of the transmission peak can be observed from 1200 to 1450 nm when the average size of the nano-holes in the perforated aluminum film increases from 400 to 500 nm (see Figure 4a,b). This red shift is expected as the surface plasmonic effect in perforated metal films can be tuned by the size of the sub-wavelength holes [22,29–30]. Compared with colloidal lithography, which requires extra process steps to charge the substrate and the colloidal spheres, metal PBL brings a more convenient way to fabricate nano-hole arrays in various kinds of metal films with the potential to be applied on the square meter scale.

### Hole density in perforated films

As shown in Figure 4c and Figure 4d, the hole density of the perforated Al film which was fabricated with 3:7 PS/PMMA solution (mass ratio) is about 400 holes/225 μm^2^ (180 million/cm^2^) and for film fabricated with 4:6 PS/PMMA solution is about 300 holes/225 μm^2^ (130 million/cm^2^). Another sample, a perforated Cr film (see Figure 2), shows a hole density of 220 million/cm^2^.

### Perspectives

The metal nano-disc arrays have a variety of potential applications such as the localized growth of ZnO nano wires or rods [31–32] or plasmonic applications [23]. The surface of the metal can be selectively modified with molecules such as thiols and used for immobilization of biomaterials [33]. The perforated metal films, which can be fabricated on large areas based on spin-coating and physical vapor deposition (PVD), have the potential to be used as wavelength-selective optical filters. In the case of our examples they let NIR light pass and block photons with longer wavelengths. It is also possible to combine the perforated metal films with micelle lithographic technique [34–35] to implant gold dots with diameter of 10 nm into the holes to form hierarchical micro–nano structures for applications such as cell-adhesion studies [36–37]. Metals like Cr, Au or Cu are good etching resists. Therefore the metal masks could be used to amplify the topographic contrast by anisotropic etching into the substrate with techniques such as reactive ion etching [38–39].

## Conclusion

Polymer blend lithography (PBL) makes use of the purely lateral phase separation of two immiscible polymers during spin-casting at controlled humidity. A metal copy of either of the polymer components can be fabricated by the selective dissolution of one component, followed by metal deposition and the final lift-off of the lithographic mask. Metal PBL starts with spin-coating of a polymer blend (PS/PMMA in MEK) film onto a substrate. A purely lateral phase separation occurs at the selected adequate conditions. Either of the components can be selectively dissolved and the other used as a lithographic mask. After the evaporation of metal and the lift-off process by snow jet treatment, a negative metal copy of the polymer mask is achieved.

The diameter of the metal islands or the holes in the metal films can be tuned by changing the molecular weight of polystyrene [21]. The typical diameter ranges from 200 to 800 nm with an average of about 400 nm (achieved by using 96 kg/mol PS and PS/PMMA mass ratio of 3:7). By using PS with a molar mass of 9.56 kg/mol, we obtained nano-islands and nano-holes with diameters ranging from 50 to 250 nm. Both the island arrays and the perforated films could be used for various applications, e.g., in cell adhesion studies, for the immobilization of biomaterials, for plasmonics such as optical filters or as resist layers for anisotropic reactive ion etching. The wavelength-selective optical transmission of our perforated films due to the localized surface plasmonic resonance was clearly demonstrated. In general, metal polymer blend lithography (metal PBL) is a new up-scalable and time-efficient method for the fabrication of functional micro/nanosized metal island arrays or nano-perforated metal films.

Large area applications in the square meter range could be realized, e.g., by using slot dye coating in a roll to roll process followed by large scale metal evaporation which is already used in the food packaging industry.

## Experimental

### Polymer solution

Poly(methyl methacrylate) (PMMA) (*M*_w_ = 9.56 kg/mol PDI = 1.05) and poly(styrene) (PS) (*M*_w_ = 96 kg/mol PDI = 1.04) were purchased from Polymer Standards Service GmbH (PSS) and dissolved directly in methyl ethyl ketone (MEK, Aldrich). The combined concentration of the two polymers was 15 mg/mL and the mass ratio between PS and PMMA was 3:7. (Exception: Figure 4b,d with PS/PMMA 4:6). For fabrication of templates with sub-100 nm structures (Figure 2) PS with a molecular weight of 9.58 kg/mol (PDI = 1.03, also purchased from PSS) was used to prepare the lithographic mask. All other parameters were kept constant.

### Cleaning of Si and quartz glass substrates

Silicon substrates were used as delivered with their native oxide layer. Both silicon and quartz glass substrates were cleaned by the snow jet method [21]: The wafers were exposed to a jet of CO_2_ ice crystals, which were produced by expanding CO_2_ stream through a nozzle. In this way, surface contaminants are removed either by mechanical impact or by dissolution in CO_2_.

### Preparation of a polymer blend lithographic mask (humidity)

The polymer blend films were spin-casted at a speed of ca. 1500 revolutions per minute (rpm) under a relative humidity of 40% onto silicon substrates cleaned by snow jet treatment (at least 20 s for a 2 cm × 2 cm substrate). The humidity was controlled by venting the chamber (about 1 L volume) with a mixture of nitrogen and water-saturated nitrogen (total flow rate approximately 40 standard cubic centimeters per minute (sccm)). The humidity in the chamber was measured by a hygrometer (Testo 635). For the optical transmission test, quartz glass was used as the substrate.

### Fabrication of metal islands and perforated films

For the fabrication of metal islands PS was selectively dissolved by cyclohexane, as shown in Figure 1b. Samples were rinsed in the solvent three times, each time for about 30 s with constant movement of the samples and then dried in nitrogen flow. The metal was deposited by a custom-built e-beam thermal evaporation machine. The distance between the evaporation source and the sample was about 60 cm. The thickness of the deposited metal film was measured by a quartz balance. For the deposition of gold, a Ti film with a thickness of 1.5 nm was deposited first in order to ensure the adhesion of the gold to the silicon substrate. The lift-off of PMMA on silicon substrates was done by snow-jet treatment. For a complete lift-off it is important that the CO_2_ gas cylinder is at room temperature and has a proper filling level. The lift-off process can also be done by using a good solvent as THF for the remaining polymer. This was done, e.g., for PMMA on quartz glass. These samples were processed by sonication in tetrahydrofuran (THF) (30 min).

For the fabrication of perforated metal films the PMMA was selectively dissolved by acetic acid as shown in Figure 1g. The metal film was then deposited the same way and the PS droplets were removed by snow-jet treatment for silicon substrate and by sonication in THF for quartz glass.

### Sample characterization

The metal patterns were characterized by scanning electron microscopy (SEM). SEM images were taken at 10 kV with a LEO 1530 SEM using a secondary electron detector. The SEM images of the metal islands and perforated films were taken at 10 kV using an in-lens detector while the AFM image was taken in tapping mode with a Bruker ICON and a custom-built AFM equipped with an Asylum MFP-3D controller. The transmission spectra were measured by a Varian Cary 500 Scan UV–vis spectrometer with open reference beam.
